# Deterministic networks for probabilistic computing

**DOI:** 10.1038/s41598-019-54137-7

**Published:** 2019-12-04

**Authors:** Jakob Jordan, Mihai A. Petrovici, Oliver Breitwieser, Johannes Schemmel, Karlheinz Meier, Markus Diesmann, Tom Tetzlaff

**Affiliations:** 1 Institute of Neuroscience and Medicine (INM-6) and Institute for Advanced Simulation (IAS-6) and JARA Institute Brain-Structure-Function Relationships (INM-10), Jülich Research Centre, Jülich, Germany; 2 Department of Physiology, University of Bern, Bern, Switzerland; 3 Kirchhoff Institute for Physics, Ruprecht-Karls-University Heidelberg, Heidelberg, Germany; 4 Department of Psychiatry, Psychotherapy and Psychosomatics, Medical Faculty, RWTH Aachen University, Aachen, Germany; 5 Department of Physics, Faculty 1, RWTH Aachen University, Aachen, Germany

**Keywords:** Network models, Biological physics

## Abstract

Neuronal network models of high-level brain functions such as memory recall and reasoning often rely on the presence of some form of noise. The majority of these models assumes that each neuron in the functional network is equipped with its own private source of randomness, often in the form of uncorrelated external noise. *In vivo*, synaptic background input has been suggested to serve as the main source of noise in biological neuronal networks. However, the finiteness of the number of such noise sources constitutes a challenge to this idea. Here, we show that shared-noise correlations resulting from a finite number of independent noise sources can substantially impair the performance of stochastic network models. We demonstrate that this problem is naturally overcome by replacing the ensemble of independent noise sources by a deterministic recurrent neuronal network. By virtue of inhibitory feedback, such networks can generate small residual spatial correlations in their activity which, counter to intuition, suppress the detrimental effect of shared input. We exploit this mechanism to show that a single recurrent network of a few hundred neurons can serve as a natural noise source for a large ensemble of functional networks performing probabilistic computations, each comprising thousands of units.

## Introduction

Probabilistic inference as a principle of brain function has attracted increasing attention over the past decades^1,2^. In support of a sampling-based “Bayesian-brain hypothesis”, the high *in-vivo* response variability of cortical neurons observed in electrophysiological recordings^3^ is interpreted in the context of ongoing probabilistic computation^4–8^. Simultaneously, it has been found that intrinsically stochastic neural networks are a suitable substrate for machine learning^9,10^. These findings have led to the incorporation of noise into computational neuroscience models^11–13^, in particular to give account for the mechanisms underlying stochastic computing such as sampling-based probabilistic inference in biological neuronal substrates^7,14–16^. Note that the term “stochastic computing” refers to the idea that the variability required for this form of computing can be mathematically described as (or replaced by) quasi-stochasticity without altering the functionality of the network. It does not imply that its implementation is relying on truly stochastic sources of noise, neither in natural nor synthetic neuronal substrates.

**Figure 1:**
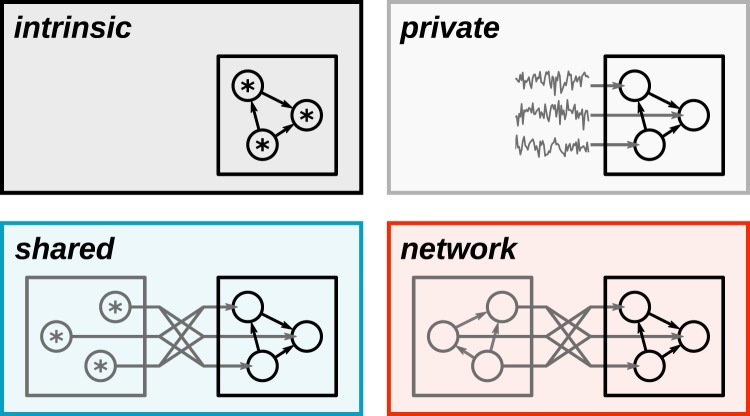
Sources of noise (gray) for functional neural networks (black). Stars indicate intrinsically stochastic units. Open circles correspond to deterministic units. Intrinsic: Intrinsically stochastic units updating their states with a probability determined by their total synaptic input. Private: Deterministic units receiving private additive independent noise. Shared: Deterministic units receiving noise from a finite population of independent stochastic sources. Network: Deterministic units receiving quasi-random input generated by a finite recurrent network of deterministic units.

A number of potential sources of noise in biological circuits have been discussed in the past^17^, such as variability in synaptic transmission^18^, ion channel noise^19^ or synaptic background input^20,21^. Arguably most widespread is the implementation of noise in neural-network models at the level of individual neurons. In this view, neurons are described as intrinsically stochastic units (Fig. 1, intrinsic) updating their states as a stochastic function of their synaptic input^14,22,23^. This description is however at odds with experimental data. *In vitro*, isolated neurons exhibit little response variability^20,24,25^. Researchers have reconciled the apparent discrepancy by equipping deterministic model neurons with additive private independent noise (Fig. 1, private), often in the form of Gaussian white noise or random sequences of action potentials (spikes) modeled as Poisson point processes^15,26^. This restores the variability required for stochastic computing and is justified as originating from the background input a neuron in nature receives from the remainder of the network. So far, it is unclear though how the biological substrate could provide such a well controlled source of stochasticity for each individual unit in the functional network. The implicit assumption of independence of the background noise across units in the network is usually mentioned en passant and goes unchallenged.

Previous experimental and theoretical studies have shown that cortical neuronal networks can generate highly irregular spiking activity with small spatial and temporal correlations, resembling ensembles of independent realizations of Poisson point processes^3,27–29^. It is hence tempting to assume that such networks may serve as appropriate effective noise sources for functional networks performing stochastic computing. However, as the size of these noise-generating background networks is necessarily finite, units in the functional network have to share noise sources. Assuming that the background spiking activity is uncorrelated, these shared inputs give rise to correlations in the inputs of the functional units, thereby violating the assumption of independence and potentially impairing network performance.

The present work demonstrates that a finite ensemble of uncorrelated noise sources (Fig. 1, shared) indeed leads to a substantial degradation of network performance due to shared-noise correlations. However, replacing the finite ensemble of uncorrelated noise sources by a recurrent neuronal network (Fig. 1, network) alleviates this problem. As shown in previous studies^30,31^, networks with dominant inhibitory feedback can generate small residual spatial correlations in their activity which counteract the effect of shared input. We propose that biological neuronal networks exploit this effect to supply functional networks with nearly uncorrelated noise despite a finite number of background inputs. Moreover, a similar noise-generation strategy may prove useful for the implementation of sampling-based probabilistic computing on large-scale neuromorphic platforms^32,33^.

In this study, we focus on neuronal networks derived from Boltzmann machines^22^ as representatives of stochastic functional networks. Such networks are widely used in machine learning^9,10^, but also in theoretical neuroscience as models of brain dynamics and function^14,15,34^. For the purpose of the present study, the advantage of models of this class lies in our ability to quantify their functional performance when subject to limitations in the quality of the noise.

## Results

### Networks with additive private Gaussian noise approximate Boltzmann machines

Boltzmann machines (BMs) are symmetrically connected networks of intrinsically stochastic binary units^22^. With an appropriate update schedule and parametrization, the network dynamics effectively implement Gibbs sampling from arbitrary Boltzmann distributions^35^. A given network realization leads to a particular frequency distribution of network states. Efficient training methods^9,36^ can fit this distribution to a given data distribution by modifying network parameters, thereby constructing a generative model of the data. In the following we investigate to what extent the functional performance of BM-like stochastic networks is altered if the intrinsic stochasticity assumed in BMs is replaced by private, shared or network -generated noise (Fig. 1). If not otherwise indicated, we consider BMs with random connectivity not trained for a specific task. Due to the specific noise-generation processes, the neural network implementations deviate from the mathematical definition of a BM. We therefore refer to these implementations as “sampling networks”.

In BMs, the intrinsically stochastic units *i* ∈ {1, …, *M*} are activated according to a logistic function ${F}_{i}({h}_{i})={(1+{e}^{-\beta {h}_{i}})}^{-1}$ of their input field ${h}_{i}={\sum }_{j\mathrm{=1}}^{M}{w}_{ij}{s}_{j}+{b}_{i}$ with inverse temperature *β*, synaptic weight *w*_*ij*_ between unit *j* and unit *i*, presynaptic activity *s*_*j*_ ∈ {0, 1}, and bias *b*_*i*_ (details see Methods). Equivalently, the network nodes of a BM may be regarded as deterministic units with Heaviside activation function *F*_*i*_(*h*_*i*_) = Θ(*h*_*i*_ + *ξ*_*i*_), receiving additive noise *ξ*_*i*_ distributed according to $\frac{\beta }{4}\mathrm{[1}-{\tanh }^{2}(\beta {\xi }_{i})]$ (^37^, see also Methods).

Additive Gaussian noise ${\xi }_{i} \sim {\mathscr{N}}(\mu ,{\sigma }^{2})$ constitutes a more plausible form of noise as it emerges naturally in units receiving a large number of inputs from uncorrelated sources^24,25,38^. Deterministic units receiving private Gaussian noise resemble units with a probabilistic update rule. Their effective gain function, however, corresponds to a shifted error function ${F}_{i}({h}_{i})={\rm{erfc}}({h}_{i}+{\mu }_{i}/\sqrt{2{\sigma }^{2}})\mathrm{/2}$, rather than a logistic function. We minimize the mismatch between the two activation functions by relating the standard deviation *σ* of the Gaussian noise to the inverse temperature *β* (see Methods). For a given noise strength, this defines an effective inverse temperature *β*_eff_. To emulate a BM at inverse temperature *β*, we rescale all weights and biases: *b*_*i*_ → *β*/*β*_eff_*b*_*i*_ − *μ*_*i*_, *w*_*ij*_ → *β*/*β*_eff_*w*_*ij*_. The Kullback-Leibler divergence *D*_KL_(*p*, p^*^) between the empirical state distribution *p* of the sampling network and the state distribution p^*^ generated by a BM over a subset of *m* units quantifies the sampling error.

**Figure 2:**
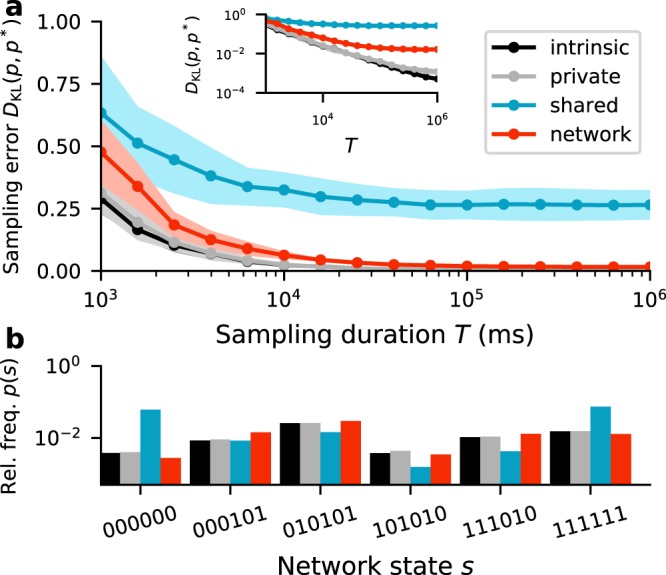
(**a**) Sampling error as measured by Kullback-Leibler divergence *D*_KL_(*p*, p^*^) between the empirical state distribution *p* of a sampling network and the state distribution p^*^ generated by the corresponding Boltzmann machine as a function of the sampling duration *T* for different sources of noise (legend, cf. Fig. 1). Error bands indicate mean ± SEM over 5 random network realizations. Inset: Same data as main panel in double-logarithmic representation. (**b**) Relative frequencies (vertical, log scale) of six exemplary states *s* (horizontal) for *T* = 10^6^ ms. Parameters: *β* = 1, *M* = 100, *K* = 200, *N* = 222, *m* = 6 (for details, see Supplementary Material).

For matched temperature, networks of deterministic units with additive Gaussian noise closely approximate BMs (Fig. 2, gray vs. black). The sampling error decreases as a function of the sampling duration *T*, and saturates at a small but finite value (Fig. 2a, gray) due to remaining differences in the activation functions and hence sampling dynamics. The residual differences between the stationary distributions (Fig. 2b, black vs. gray bars) are significantly smaller than the differences in relative probabilities of different network states.

The assumption of idealized private Gaussian noise generated by pseudorandom number generators is hard to reconcile with biology. In the following, the Gaussian noise is therefore replaced by input from binary and, subsequently, spiking units, thereby mimicking an embedding of the functional circuit into a cortical network. As a consequence, the noise of the sampling units exhibits jumps with finite amplitudes determined by the weights of the incoming connections. Only if the number of input events per sampling unit is large and the weights are small, the collective signal resembles Gaussian noise (see Supplementary Material). The sampling error resulting from private Gaussian noise therefore constitutes a lower bound on the error achievable by sampling networks supplied with noise from a finite ensemble of binary or spiking sources.

### Shared-noise correlations impair sampling performance

**Figure 3:**
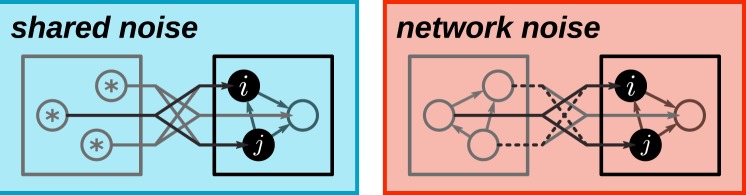
Origin of shared-input correlations and their suppression by correlated presynaptic activity. A pair of neurons *i* and *j* receiving input from a finite population of noise sources (left) or a recurrent network (right). The input correlation ${C}_{ij}^{{\rm{in}}}$ decomposes into a contribution ${C}_{{\rm{shared}},ij}^{{\rm{in}}}$ resulting from shared noise sources (solid black lines) and a contribution ${C}_{{\rm{corr}},ij}^{{\rm{in}}}$ due to correlations between sources (dashed black lines). If Dale’s law is respected (neurons are either excitatory or inhibitory), shared-input correlations are always positive (${C}_{{\rm{shared}},ij}^{{\rm{in}}} > 0$). Left: In the shared -noise scenario, sources are by definition uncorrelated (${C}_{{\rm{corr}},ij}^{{\rm{in}}}=0$) and cannot compensate for shared-input correlations. Right: In inhibition-dominated neural networks (network case), correlations between units arrange such that ${C}_{{\rm{corr}},ij}^{{\rm{in}}}$ is negative, thereby compensating for shared-input correlations such that the total input correlation ${C}_{ij}^{{\rm{in}}}$ approximately vanishes.

Neurons in a functional circuit embedded in a finite surrounding network have to share noise sources to gather random input at a sufficiently high frequency. In consequence, the input noise for pairs of sampling units is typically correlated, even (or, in particular) if the noise sources are independent (Fig. 3, left).

By replacing private noise with a large number of inputs from a finite ensemble of independent noise sources, we investigate to what extent these shared-noise correlations distort the sampled distribution of network states. The noise sources are stochastic binary units with an adjustable average activity 〈z〉. To achieve a high input event count, each sampling unit is randomly assigned a large number *K* of inputs. For each unit, these are randomly chosen from a common ensemble of *N* sources. On average, a pair of neurons in the sampling network hence shares *K*^2^/*N* noise sources. The ensemble of noise sources is comprised of *γN* excitatory and (1 − *γ*)*N* inhibitory units, projecting to their targets with weights *w* and −*gw*, respectively. The input field for a single unit in the sampling network is then given by ${h^{\prime} }_{i}={\sum }_{j=1}^{M}\,{w}_{ij}{s}_{j}+{b}_{i}+{\sum }_{k=1}^{N}\,{m}_{ik}{z}_{k}$, where *m*_*ik*_ represents the strength of the connection from the *k* th noise source to the *i* th sampling unit.

For homogeneous connectivity, i.e., identical input statistics for each sampling unit, the second term in ${h^{\prime} }_{i}$ can be approximated by a Gaussian noise with mean *μ* = *Kw*(*γ* − (1 − *γ*)*g*)〈z〉 and variance *σ*^2^ = *Kw*^2^(*γ* + (1 − *γ*)*g*^2^)〈z〉(1 − 〈*z*〉) (see Methods). These measures allow us to perform a similar calibration of the activation function as in the previous section. For heterogeneous connectivity, a similar calibration can be performed based on the empirically obtained mean and variance of the noise input distribution.

**Figure 4:**
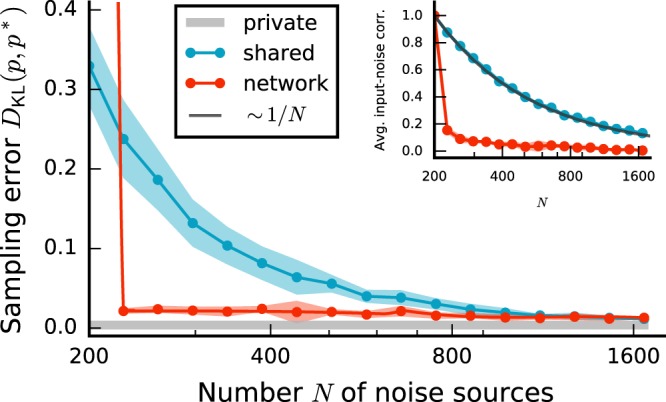
Sampling error *D*_KL_(*p*, p^*^) as a function of the number *N* of noise sources for different sources of noise (legend). Error bands indicate mean ± SEM over 5 random-network realizations. Inset: Dependence of average input correlation coefficient *ρ* of mutually unconnected sampling units on *N*. Black curve represents $\sim \mathrm{1/}N$ fit. Sampling duration *T* = 10^5^ ms. Remaining parameters as in Fig. 2.

If *K* ≈ *N*, shared-input correlations are large and the sampling error is substantial, even for long sampling duration (Fig. 2, blue curve and bars). Increasing *N* while keeping *K* fixed leads to a gradual decrease of shared-input correlations (~1/*N*) and therefore to a reduction of the sampling error (Fig. 4, blue curves). For large *N* ≫ *K*, the sampling error approaches values comparable to those obtained with private Gaussian noise (Fig. 4, blue vs. gray curves). For a broad range of *N*, the sampling error and the average shared-input correlation exhibit a similar trend (~1/*N*).

### Network-generated noise recovers sampling performance

In recurrent neural networks, inhibitory feedback naturally suppresses shared-input correlations through the emerging activity patterns^30,31^. Here we exploit this effect to minimize the detrimental influence of shared-input correlations arising from a limited number of noise sources. To this end, we replace the finite ensemble of independent stochastic sources by a recurrent network of deterministic units with Heaviside activation function(Fig. 1, red; see Methods). The noise generating network comprises an excitatory and an inhibitory subpopulation with random, sparse and homogeneous connectivity. Connectivity parameters are chosen such that the recurrent dynamics is dominated by inhibition, thereby guaranteeing stable, nearly uncorrelated activity^27,30,39,40^. To achieve optimal suppression of shared-input correlations in the sampling network, the connectivity between the noise network and the sampling network needs to match the connectivity within the noise network, i.e. the number and the (relative) weights of excitatory and inhibitory inputs have to be identical. Similar to the previous sections, we map the sampling network to a corresponding BM by relating the noise intensity to the inverse temperature *β*. As above, the additional contribution to the input fields ${h^{\prime} }_{i}$ of neurons in the sampling network resulting from the noise network can be approximated by a normal distribution ${\mathscr{N}}(\mu ,{\sigma }^{2})$. Here, we account for an additional contribution to the input variance resulting from residual correlations between units in the noise network (see Supplementary Material).

Using a recurrent network for noise generation considerably decreases the sampling error compared to the error obtained with a finite number of independent sources (shared-noise scenario), even if the shared-input correlations are substantial (Fig. 2, red vs. blue curve). Precisely because the activity of the noise network is not uncorrelated, the shared-input correlations in the units of the functional circuit are counterbalanced (cf. Fig. 3). For a broad range of noise-network sizes *N*, the noise input correlation, and hence the sampling error, are significantly reduced (Fig. 4, red vs. blue). In this range, the sampling error is comparable to the error obtained with private Gaussian noise and almost independent of *N* (Fig. 4, red vs. gray). Only if the noise network becomes too dense (*K* ≈ *N*), its dynamics lock into a fixed point (see Supplementary Material) and the sampling performance breaks down.

At first glance, it may seem counterintuitive that correlated network-generated noise can suppress correlations resulting from shared input. To resolve this, consider the noise input correlation

1 $${C}_{ij}^{{\rm{in}}}=\langle \sum _{k\in  {\mathcal B} }\,{m}_{ik}{\bar{z}}_{k}\sum _{l\in  {\mathcal B} }\,{m}_{jl}{\bar{z}}_{l}\rangle =\mathop{\underbrace{\sum _{k\in  {\mathcal B} }\,{m}_{ik}{m}_{jk}\langle {\bar{z}}_{k}^{2}\rangle }}\limits_{{C}_{{\rm{shared}},ij}^{{\rm{in}}}}+\mathop{\underbrace{\sum _{k\in  {\mathcal B} }\,\sum _{l\in  {\mathcal B} }\mathrm{(1}-{\delta }_{kl}){m}_{ik}{m}_{jl}\langle {\bar{z}}_{k}{\bar{z}}_{l}\rangle }}\limits_{{C}_{{\rm{corr}},ij}^{{\rm{in}}}}$$

of two units *i* and *j* in the (unconnected) sampling network. Here, ${\bar{z}}_{k}={z}_{k}-\langle {z}_{k}\rangle$ denotes the centered activity of unit *k* in the pool ${\mathcal B}$ of noise sources, *m*_*ik*_ the weight of the connection between noise unit *k* and the target un*i*t *i*, 〈·〉 the trial average (average across different initial conditions), and *δ*_*kl*_ the Kronecker delta. The first term ${C}_{{\rm{shared}},ij}^{{\rm{in}}}$ in (1) describes shared-input correlations arising from common noise sources. The second term ${C}_{{\rm{corr}},ij}^{{\rm{in}}}$ represents pairwise correlations between noise sources (Fig. 3; see also Eq. (19) in^31^). If Dale’s principle is respected, i.e., if the weights *m*_*ik*_ from a given noise source *k* have identical sign for all targets *i*, the first contribution is always positive. In the shared-noise scenario, ${C}_{{\rm{corr}},ij}^{{\rm{in}}}$ is zero, since, by definition, the sources are uncorrelated. In this case, the average noise input correlation is solely determined by the connectivity statistics (see inset in Fig. 4, compare dark gray and blue). In the network-noise scenario, in contrast, the sources are not uncorrelated due recurrent interactions in the noise network. As shown in^30,31^, ${C}_{{\rm{corr}},ij}^{{\rm{in}}}$ is negative in balanced inhibition-dominated recurrent neuronal networks (both in purely inhibitory and in excitatory-inhibitory networks) and nearly cancels the contribution ${C}_{{\rm{shared}},ij}^{{\rm{in}}}$ of shared inputs, such that the total input correlation ${C}_{ij}^{{\rm{in}}}$ is close to zero. Shared components of the input fluctuations are canceled by inhibitory feedback, resulting in nearly uncorrelated inputs despite substantial overlap in the presynaptic populations. Here, we exploit exactly this effect: a network in the balanced state supplies noise with a correlation structure that suppresses shared-input correlations. As noise input correlations are decreased, the performance of the sampling networks is increased (Fig. 4).

### Deterministic neural networks serve as a suitable noise source for a model of handwritten-digit generation

All realizations within the ensemble of unspecific, randomly generated sampling networks considered so far exhibit consistent performance characteristics (cf. narrow error bands in Figs. 2 and 4). Here, we demonstrate similar behaviour for a sampling network where the weights and biases are not chosen randomly but trained for a specific task – the generation of handwritten digits with imbalanced class frequencies (see Methods). Since it is not possible to measure the state distribution over all units in the network (2^786+10^ states), we restrict the analysis to the states of label units as a compressed representation of the full network states. Training is performed using ideal Boltzmann machines. Weights and biases are calibrated as before. Noise is added to the training samples to reduce overfitting and thereby improve mixing performance. To make the task more challenging, the Boltzmann machine is trained to generated odd digits twice as often as even digits (see Methods).

**Figure 5:**
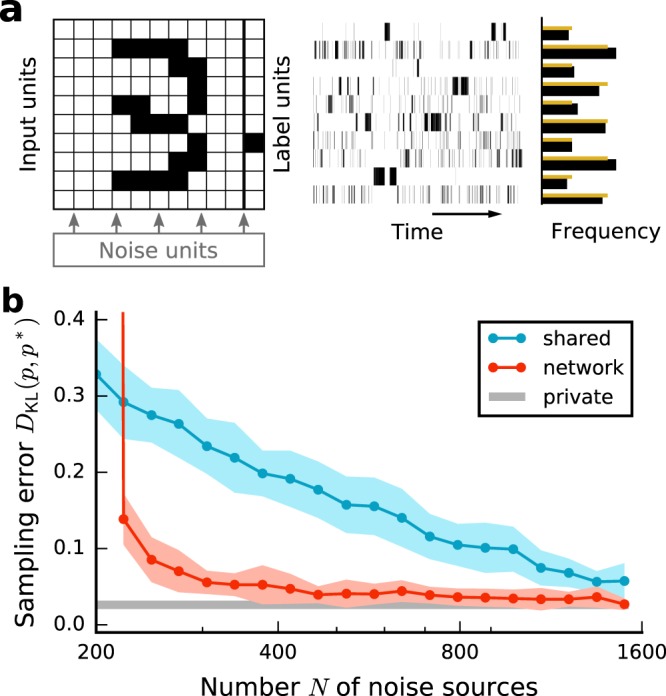
Performance of a generative network trained on an imbalanced subset of the MNIST dataset for different noise sources (legend). (**a**) Left: Sketch of the network consisting of external noise inputs, input units, trained to represent patterns corresponding to handwritten digits, and label units trained to indicate the currently active pattern. Right: Network activity and trial-averaged relative activity of label units for intrinsic noise (black) and target distribution (yellow), with even digits occurring twice as often as odd digits. (**b**) Sampling error *D*_KL_(*p*, p^*^) between the empirical state distribution *p* of label units and the state distribution p^*^ of label units generated by the corresponding Boltzmann machine as a function of the number *N* of noise sources for shared and network case. Error bands indicate mean ± SEM over 20 trials with different initial conditions and noise realizations.

The results are similar to those obtained for sampling networks with random weights and biases: (i) networks with private external noise perform close to optimal, (ii) shared noise correlations impair network performance, and (iii) the performance is restored by employing a recurrent network for noise generation (Fig. 5). Thus, deterministic recurrent neural networks qualify as a suitable noise source for practical applications of neural networks performing probabilistic computations.

### Shared-input correlations impair network performance for high-entropy tasks

The dynamics of a BM representing a high-entropy distribution evolve on a flat energy landscape with shallow minima, resulting in small pairwise correlations between sampling units. Here, the sampling process is sensitive to perturbations in statistical dependencies, such as those caused by shared-input correlations. In contrast, the sampling dynamics in BMs representing low-entropy distributions with pronounced peaks are dominated by deep minima in the energy landscape. In this case, correlations between sampling units are typically large and noise input correlations have little effect.

**Figure 6:**
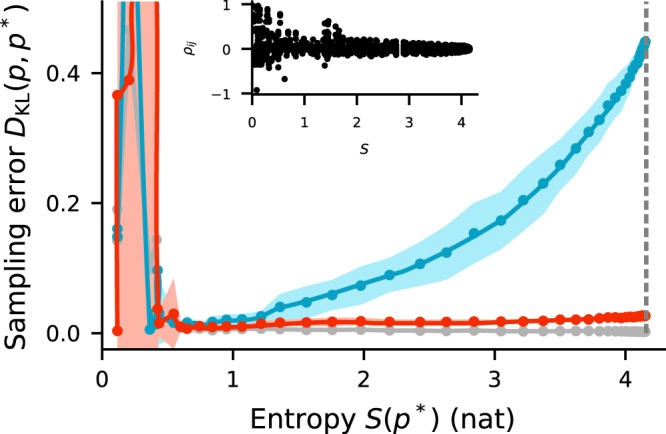
Sampling error *D*_KL_(*p*, p^*^) as a function of the entropy *S* of the target distribution for different sources of noise (same colors as in other figures). Error bands indicate mean ± SEM over 5 random network realizations. Vertical dashed gray line indicates maximal entropy, corresponding to a uniform target distribution. Inset: pairwise activity correlation coefficients in a Boltzmann machine for different entropies of the sampled state distribution. Sampling duration *T* = 10^5^ ms. Remaining parameters as in Fig. 2.

We systematically vary the entropy of the target distribution by changing the inverse temperature *β* in a BM and adjusting the relative noise strength in the other cases accordingly (Fig. 6). Since *β* always appears as a multiplicative factor in front of weights and biases, this is equivalent to scaling weights and biases globally. For small entropies, the sampling error for shared and network noise is comparable to the error obtained with private noise, despite substantial shared-input correlations. Consistent with the intuition provided above, the sampling error for shared noise increases significantly with increasing entropy, whereas in the other cases it remains low.

We conclude that generally the effect of shared-noise correlations on the functional performance of sampling networks depends on the entropy of the target distribution, or, equivalently, on the absolute magnitude of functional correlations between sampling units. For high-entropy tasks, such as pattern generation, shared-input correlations can be highly detrimental. For low-entropy tasks, such as pattern classification, they presumably play a less significant role. Nevertheless, independent of the entropy of the task, functional performance for network-generated noise is close to optimal.

### Small recurrent networks provide large sampling networks with noise

Both from a biological as well as a technical point of view, it makes sense to minimize material and energy costs for noise generation. To achieve a good sampling performance, the number *N* of noise sources as well as the number *K* of noise inputs per functional unit need to be sufficiently large (Fig. 4). Therefore, a certain minimal amount of resources have to be reserved for noise generation. However, once these resources are allocated, small recurrent networks can provide noise for large sampling networks without sacrificing computational performance. We note in passing that, moreover, a single noise network can supply an arbitrary number of independent functional networks with noise.

**Figure 7:**
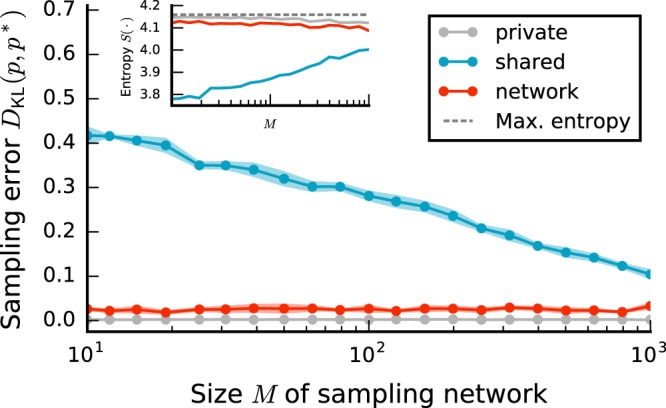
Sampling error *D*_KL_(*p*, p^*^) as a function of the sampling-network size *M* for different sources of noise (legend). Error bands indicate mean ± SEM over 5 random network realizations. Inset: Entropy of the sampled state distribution *p* as a function of *M*. Horizontal dashed dark gray line indicates entropy of uniform distribution, i.e., maximal entropy. Average weight in sampling networks: ${\mu }_{{\rm{BM}}}=-\,\mathrm{0.15/}\sqrt{M}$. Sampling duration *T* = 10^5^ ms. Remaining parameters as in Fig. 2.

Here, we vary the size of the sampling network *M*, while keeping *N* and the number *m* of observed neurons fixed (Fig. 7). As the variance of the input distribution for a neuron in the sampling network scales proportionally to its in-degree, and the sampling network is fully connected, increasing *M* reduces the effective noise amplitude. As a consequence, the entropy of the marginal distribution over the subset of observed neurons changes (see Supplementary Material), thereby influencing the sampling performance in the presence of shared-noise correlations (see previous section). To avoid this effect, we scale the weights in the sampling network with $\mathrm{1/}\sqrt{M}$^30,39,41^, thereby keeping the entropy of the marginal target distribution approximately constant (Fig. 7 inset, gray curve).

In the presence of private noise, the sampling error is small and independent of *M* (Fig. 7). As before, the performance is considerably impaired for shared noise. The decrease in the error for larger sampling networks cannot be traced back to a change in entropy, by virtue of the weight scaling. Instead, the decrease results from a more efficient suppression of external correlations within the sampling network arising from the growing negative feedback for increasing *M* in sampling networks with net recurrent inhibition^39^. Still, even for large *M*, the error remains significantly larger than the one obtained with private noise. For network noise, in contrast, the error is almost as small as for private noise, and independent of *M*. Qualitatively similar findings are also obtained without scaling synaptic weights unless the entropy of the target distribution is too small (details see Supplementary Material).

### Networks of spiking neurons implement neural sampling without noise

**Figure 8:**
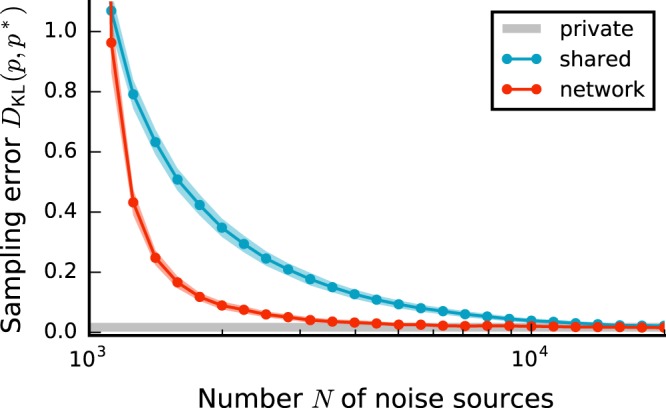
Sampling in spiking networks with biologically plausible noise networks. Kullback-Leibler divergence *D*_KL_(*p*, p^*^) between the empirical state distribution *p* of a sampling network of spiking neurons and the state distribution p^*^ generated by the corresponding Boltzmann machine as a function of the number *N* of noise sources. Error bands indicate mean ± SEM over 10 random network realizations (see Supplementary Material).

The results so far rest on networks of binary model neurons. Their dynamics are well understood^30,34,39–41^, and their mathematical tractability simplifies the calibration of sampling-network parameters for network-generated noise. Neurons in mammalian brains communicate, however, predominantly via short electrical pulses (spikes). It was shown previously^15,42^, that networks of spiking neurons with private external noise can approximately represent arbitrary Boltzmann distributions, if binary-unit parameters are properly translated to spiking-neuron parameters (see gray curve in Fig. 8; see Methods and Supplementary Material).

Consistent with our results on binary networks, the sampling performance of networks of spiking leaky integrate-and-fire neurons decreases in the presence of shared-noise correlations, but recovers for noise provided by a recurrent network of spiking neurons resembling a local cortical circuit with natural connection density and activity statistics (Fig. 8). Similar to binary networks, a minimal noise-network size *N* ensures an asynchronous activity regime, a prerequisite for good sampling performance. Spiking noise networks that are too densely connected (*K*/*N* → 1) tend to synchronize, causing large sampling errors (see red curve in Fig. 8 for small *N*).

## Discussion

Consistent with the high variability in the activity of biological neural networks^17^, many models of high-level brain function rely on the presence of some form of noise. We propose that additive input from deterministic recurrent neural networks serves as a well controllable source of noise for functional network models. The article demonstrates that networks of deterministic units with input from such noise-generating networks can approximate a large variety of target distributions and perform well in probabilistic generative tasks. This scheme covers both networks of binary and networks of spiking model neurons, and leads to an economic usage of resources in biological and artificial neuromorphic systems.

From a biological perspective, our concept is tightly linked to experimental evidence. In the absence of synaptic input (*in vitro*), fluctuations in the membrane potentials of single neurons are negligible. Consequently, the variability in neuronal *in-vitro* responses is small^20,24,25^. In the presence of synaptic inputs from an active surrounding network (*in vivo*), in contrast, fluctuations in membrane potentials are substantial and the response variability is large^3^. Furthermore, biological neural networks exhibit an abundance of inhibitory feedback connections. The active suppression of shared-input correlations by inhibitory feedback^30,31^, i.e., the mechanism underlying the present work, accounts for the small correlations in the activity of pairs of neurons observed *in vivo*^29^. Moreover, the theory correctly describes the specific correlation structure of inputs observed in pairwise *in-vivo* cell recordings^43^. Hence, active decorrelation via inhibitory feedback shapes the *in-vivo* activity. Here, we propose a functional role for this decorrelation mechanism: cortical circuits supply each other with quasi-uncorrelated noise. Note that a similar mechanism for variability injection has been hypothesized to lie at the basis of song learning in Zebra finches: a cortical-basal ganglia loop actively generates variability necessary for successful motor learning^44,45^.

For conceptual simplicity, the study segregates a neuronal network into a functional and a noise-generating module. In biological substrates, these two modules may be intermingled. The noise-generating module may be interpreted as an ensemble of distinct functional networks serving as a “heat bath” for a specific functional circuit. In this view, one network’s function is another network’s noise.

We show that shared-noise correlations can be highly detrimental for sampling from given target distributions. Generating noise with recurrent neural networks overcomes this problem by exploiting active decorrelation in networks with inhibitory feedback^30,31^. As an alternative solution, the effect of shared-input correlations could be mitigated by training functional network models in the presence of these correlations^46^. However, this approach is specific to particular network models. Moreover, it prohibits porting of models between different substrates. Networks previously trained under specific noise conditions will not perform well in the presence of noise with a different correlation structure. Our approach, in contrast, constitutes a general-purpose solution which can also be employed for models that cannot easily be adapted to the noise statistics, such as hard-wired functional network models^26,47^ or bottom-up biophysical neural-network models^48,49^.

In biological neural networks, the probabilistic gating of ion channels in the cell membrane^19^ and the variability in synaptic transmission^18^ constitute alternative potential sources of stochasticity. However, for the majority of stochastic network models, ion-channel noise is too small to be relevant: in the absence of (evoked or spontaneous) synaptic input, fluctuations in membrane potentials recorded *in vitro* are in the *μ*V range and hence negligible compared to the mV fluctuations necessary to support sampling-based approaches^15^. Synaptic stochasticity has been studied both *in vitro*^18,50,51^ and *in vivo*^52,53^ and comes in two distinct forms: spontaneous release and variability in evoked postsynaptic response amplitudes, including synaptic failure. The rate of spontaneous synaptic events measured at the soma of the target neuron is in the range of a few events per second^54,55^. The resulting fluctuations in the input are therefore negligible. The variability in postsynaptic response amplitudes, in contrast, is substantial and can have multiple origins: in the absence of background activity (*in vitro*), response amplitudes vary due to a quasi-stochastic fusion of vesicles with the presynaptic membrane and release of neurotransmitter. *In vivo*, other complex deterministic processes such as the interplay between background input and short-term plasticity, the voltage dependence of synaptic currents or shunting may further contribute to this form of quasi-stochasticity. The variability in postsynaptic response amplitudes has often been suggested as a plausible noise resource for computations in neural circuits^16,56–60^. Due to its multiplicative, state-dependent nature, this form of noise is fundamentally different from the additive noise usually employed in sampling models. Neftci *et al*.^16^ propose a model of stochastic computation in neuronal substrates employing a specific model of synaptic stochasticity. Due to the state-dependent nature of noise generated by stochastic synapses, the resulting systems do not resemble Boltzmann machines in general. The authors nevertheless demonstrate that such networks can be trained to classify handwritten digits with contrastive divergence, a learning algorithm specific to Boltzmann machines. Apart from this specific experimental demonstration, the authors do not provide any systematic analysis of their model. In particular, it remains unclear why and under what conditions contrastive divergence is a suitable learning algorithm. A theoretically solid model of sampling-based computations in neuronal substrates employing synaptic stochasticity as a noise resource remains a topic for future studies.

The present work focuses on a specific class of neuronal networks performing sampling-based probabilistic inference. An alternative approach to sampling-based Bayesian computation in neural circuits is provided by models relying on a parametric instead of a sample-based representation of probability distributions^5,61–63^. In contrast to the methods considered here, the posterior distributions are computed essentially instantaneously without requiring the collection of samples. Such a parametric approach however comes at the cost of restricting the distributions that can be represented by a particular network architecture. In addition, learning in these systems remains a topic of ongoing research, while powerful learning algorithms exist for networks performing sampling-based inference^36^.

Some neuromorphic-hardware systems follow innovative approaches to the generation of uncorrelated noise for stochastic network models, such as exploiting thermal noise and trial-to-trial fluctuations in neuron parameters^64–66^. However, hardware systems need to be specifically designed for a particular technique and sacrifice chip area that otherwise could be used to house neurons and synapses. The solution proposed in this article does not require specific hardware components for noise generation. It solely relies on the capability of emulating recurrent neural networks, the functionality most neuromorphic-hardware systems are designed for. On the analog neuromorphic system Spikey^67^, for example, it has already been demonstrated that decorrelation by inhibitory feedback is effective and robust, despite large heterogeneity in neuron and synapse parameters and without the need for time-consuming calibrations^68^. While a full neuromorphic-hardware implementation of the framework proposed here is still pending, the demonstration on Spikey shows that our solution is immediately implementable and feasible.

## Methods

### Binary network simulation

Sampling networks consist of *M* binary units that switch from the inactive (0) to the active (1) state with a probability *F*_*i*_(*h*_*i*_) := *p*(*s*_*i*_ = 1|*h*_*i*_), also referred to as the “activation function”. The input field *h*_*i*_ of a unit depends on the state of the presynaptic units and is given by:

2 $${h}_{i}({\rm{s}})=\sum _{j}\,{w}_{ij}{s}_{j}+{b}_{i}.$$

Here *w*_*ij*_ denotes the weight of the connection from unit *j* to unit *i* and *b*_*i*_ denotes the bias of unit *i*. We perform an event-driven update, drawing subsequent inter-update intervals *τ*_*i*_ ~ Exp(*λ*) for each unit from an exponential distribution with rate *λ* := 1/*τ* with an average update interval *τ*. Starting from *t* = 0, we update the neuron with the smallest update time *t*_*i*_, choose a new update time for this unit *t*_*i*_ + *τ*_*i*_ and repeat this procedure until any *t*_*i*_ is larger than the maximal simulation duration *T*_max_. Formally, this update schedule is equivalent to an asynchronous update where a random unit is selected at every update step^22,30,39,69^. The introduction of “update times” only serves to introduce a natural time-scale of neuronal dynamics (see, e.g.^39^).

### Random sampling networks

Weights are randomly drawn from a beta distribution Beta(*a*, *b*) and shifted to have mean *μ*_BM_. We choose the beta distribution with *a* = 2, *b* = 2 as it generates interesting Boltzmann distributions while having finite support, thereby reducing the probability of generating distributions with almost isolated states. The small error across randomly chosen initial conditions in Figs. 2, 4, 6 and 7 indicates that all randomly generated sampling networks indeed possess good mixing properties, i.e., the typical time taken to traverse the state space is much smaller than the total sampling duration. Weights are symmetric (*w*_*ij*_ = *w*_*ji*_) and self connections are absent (*w*_*ii*_ = 0). To control the average activity in the network, the bias for each unit is chosen such that on average, it cancels the input from the other neurons in the network for a desired average activity 〈s〉: *b*_i_ = M*μ*〈s〉^39^. Whenever a unit is updated, the state of (a subset) of all units in the sampling network is recorded. To remove the influence of initial transients, i.e., the burn-in time of the Markov chain, samples during the initial interval of each simulation (*T*_warmup_) are excluded from the analysis. From the remaining samples we compute the empirical distribution *p* of network states. The following sections introduce the activation function for the units for different ways of introducing noise to the system.

### Intrinsic noise

Intrinsically stochastic units switch to the active state with probability

3 $${F}_{i}({h}_{i})=\frac{1}{1+{e}^{-\beta {h}_{i}}},$$

where *β* determines the slope of the logistic function and is also referred to as the “inverse temperature”. For small *β*, changes in the input field have little influence of the update probability, while for large beta a unit is very sensitive to changes in *h*_*i*_ and in the limit *β* → ∞ the activation function becomes a Heaviside step function. Symmetric networks with these single-unit dynamics and the update schedule described in Binary network simulation are identical to Boltzmann machines, leading to a stationary distribution of network states of Boltzmann form:

4 $$p({\rm{s}}) \sim \exp (\frac{\beta }{2}\sum _{i,j}\,{w}_{ij}{s}_{i}{s}_{j}+\beta \sum _{i}\,{b}_{i}{s}_{i}).$$

Instead of directly prescribing a stochastic update rule like Eq. 3, we can view these units as deterministic units with a Heaviside activation function and additive noise on the input field:

$${F}_{i}({h}_{i})=\Theta ({h}_{i}+{\xi }_{i}),$$

with ${\xi }_{i} \sim \frac{\beta }{4}\mathrm{(1}-{\tanh }^{2}(\beta {\xi }_{i}))$^37^ and Θ denoting the Heaviside step function

5 $$\Theta (x)=\{\begin{array}{cc}1 & {\rm{i}}{\rm{f}}\,x\ge 0\\ 0 & {\rm{e}}{\rm{l}}{\rm{s}}{\rm{e}}\end{array}$$

Averaging over the noise *ξ*_*i*_ yields the probabilistic update rule (Eq. 3). However, on biophysical grounds it is difficult to argue for this particular distribution of the noise.

### Private noise

We consider a deterministic model in which we assume a more natural distribution for the additive noise, namely Gaussian form (${\xi }_{i} \sim {\mathscr{N}}({\mu }_{i},{\sigma }_{i}^{2})$), for example arising from a large number of independent background inputs^38^. In this case, the noise averaged activity for fixed *h*_*i*_ is given by:

6 $$\begin{array}{rcl}{F}_{i}({h}_{i}) & = & {\int }_{-\infty }^{\infty }\,{\rm{d}}{\xi }_{i}\,\Theta ({h}_{i}+{\xi }_{i})p({\xi }_{i})\\  & = & {\int }_{-{h}_{i}}^{\infty }\,{\rm{d}}{\xi }_{i}\,{\mathscr{N}}({\mu }_{i},{\sigma }_{i}^{2})\\  & = & \frac{1}{2}{\rm{erfc}}(-\frac{{h}_{i}+{\mu }_{i}}{\sqrt{2}{\sigma }_{i}}).\end{array}$$

Similar to the intrinsically stochastic units (Intrinsic noise), the update rule for deterministic units with Gaussian noise is effectively probabilistic. Both functions share some general properties (bounded, monotonic):

$$\begin{array}{rcl}\mathop{\mathrm{lim}}\limits_{{h}_{i}\to -\infty }{F}_{i}({h}_{i}) & = & 0,\\ \mathop{\mathrm{lim}}\limits_{{h}_{i}\to \infty }{F}_{i}({h}_{i}) & = & 1,\\ {\partial }_{{h}_{i}}{F}_{i}({h}_{i}) &  >  & 0\,\forall {h}_{i},\end{array}$$

and one can hence hope to approximate the dynamics in Boltzmann machines with a network of deterministic units with Gaussian noise by a systematic matching of parameters.

One approach is to choose parameters for the Gaussian noise such that the difference between the two activation functions is minimized. To simplify notation we drop the index *i* in the following calculations. Since both activation functions are symmetric around zero, we require that their value at *h* = 0 is identical, fixing one parameter of the noise distribution (*μ* = 0). To find an expression for the noise strength *σ*, the simplest method equates the coefficients of a Taylor expansion up to linear order of both activation functions around zero. For the logistic activation function (Eq. 3) this yields:

$$F(h)=0.5+0.25\beta h+{\mathscr{O}}({h}^{2}),$$

while for the units with Gaussian noise (Eq. 6) we obtain

$$F(h)=0.5+\frac{1}{\sqrt{2\pi }\sigma }h+{\mathscr{O}}({h}^{2}\mathrm{).}$$

Equating the coefficients of *h* gives an expression for the noise strength *σ* as a function of the inverse temperature *β*:

7 $$\sigma (\beta )=\frac{2\sqrt{2}}{\sqrt{\pi }\beta }.$$

**Figure 9:**
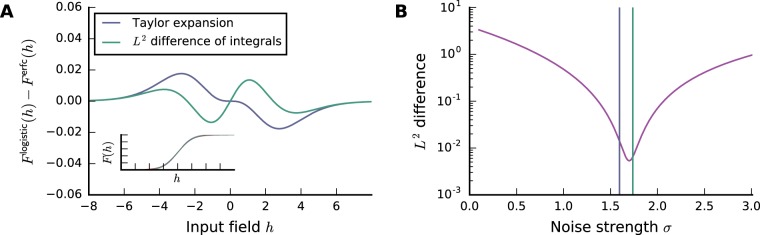
Fit of error function to logistic function via Taylor expansion (purple) and *L*^2^ difference of integrals (green). (**A**) Difference of logistic activation function and error function with adjusted *σ* via Eq. 7 (purple) and via Eq. 10 (green). Inset: activation functions. (**B**) *L*^2^ difference of activation functions (Eq. 8) as a function of the strength of the Gaussian noise *σ*. Vertical bars indicate *σ* obtained via the respective method.

While this approach is conceptually simple, the Taylor expansion around zero leads to large deviations between the activation functions for input fields different from zero (Fig. 9).

Another option taking into account all possible values of *h* is to minimize the *L*^2^ difference of the two activation functions:

8 $$\sigma =\mathop{{\rm{\arg }}\,{\rm{\min }}}\limits_{\sigma ^{\prime} }\int \,{\rm{d}}h\,{(l(h)-g(h,\sigma ^{\prime} ))}^{2},$$

where *l* denotes the logistic and *g* the activation function for Gaussian noise. Since it is not possible to analytically evaluate the resulting integral, we opt for a slightly simpler approach: minimizing the *L*^2^ difference of integrals of the activation function from −∞ to 0:

$$\sigma =\mathop{{\rm{\arg }}\,{\rm{\min }}}\limits_{\sigma ^{\prime} }{(L(h{)|}_{-\infty }^{0}-G(h,\sigma ^{\prime} {)|}_{-\infty }^{0})}^{2},$$

with capital letters denoting antiderivatives. To find the minimal *σ*, we take the derivative of the right hand side with respect to *σ*′ and equate it with zero:

$$-2(F(h{)|}_{-\infty }^{0}-G(h{)|}_{-\infty }^{0}){\partial }_{\sigma }G(h{)|}_{-\infty }^{0}=0.$$

From this we observe that

9 $$(F(h{)|}_{-\infty }^{0}-G(h{)|}_{-\infty }^{0})=0,$$

is a sufficient condition to satisfy this equation. We compute the integral of both activation functions. For the logistic activation function (Eq. 3) we obtain:

$$\begin{array}{rcl}\int \,{\rm{d}}h\,F(h) & = & \int \,{\rm{d}}h\,\frac{1}{1+{e}^{-\beta h}}\\  & = & h+\frac{\log \,\mathrm{(1}+{e}^{-\beta h})}{\beta },\end{array}$$

with the definite integral

$${\int }_{-\infty }^{0}\,{\rm{d}}h\,F(h)=\frac{\log \,2}{\beta },$$

since the two diverging terms for *h* → −∞ cancel. For the activation function with Gaussian noise (Eq. 6) we get:

$$\begin{array}{rcl}\int \,{\rm{d}}h\,F(h) & = & \int \,{\rm{d}}h\,\frac{1}{2}{\rm{erfc}}(\frac{-h}{\sqrt{2}\sigma })\\  & = & \frac{\sigma }{\sqrt{2\pi }}{e}^{-\frac{{h}^{2}}{2{\sigma }^{2}}}+0.5h{\rm{erfc}}(\frac{-h}{\sqrt{2}\sigma }),\end{array}$$

and computing the definite integral leads to:

$${\int }_{-\infty }^{0}{\rm{d}}h\,F(h)=\frac{\sigma }{\sqrt{2\pi }}\,,$$

since the second term vanishes for *h* → −∞ as the complementary error function decreases faster than |*h*|^−1^. From Eq. 9 we hence find *σ* as a function of *β*:

10 $$\sigma (\beta )=\frac{\log \,2\sqrt{2\pi }}{\beta }.$$

Even though this value is not minimizing the *L*^2^ difference, it provides a better fit than that obtained by simply Taylor expanding around zero, since in this case we are also taking into account the mismatch for larger absolute values of *h* (Fig. 9). We will hence use Eq. 10 to translate between the inverse temperature *β* of the logistic activation function and the strength *σ* of the Gaussian noise.

### Shared noise

In the previous section we have assumed that each deterministic unit in the sampling network receives private, uncorrelated Gaussian noise. Now we instead consider a second population ${\mathcal B}$ of $N=| {\mathcal B} |$ mutually unconnected, intrinsically stochastic units with logistic activation functions (cf. Intrinsic noise) that provide additional input to units in the sampling network. In the following we will denote the population/set of units in the sampling network by ${\mathscr{S}}$ and refer to the second population as the background population or noise population. The input field for a unit *i* in the sampling network ${\mathscr{S}}$ hence contains an additional term arising from projections from the background population (cf. Eq. 2):

11 $${h^{\prime} }_{i}=\mathop{\underbrace{\sum _{j\in {\mathscr{S}}}{w}_{ij}{s}_{j}+{b}_{i}}}\limits_{{h}_{i}}+\mathop{\underbrace{\sum _{k\in  {\mathcal B} }\,{m}_{ik}{z}_{k}}}\limits_{{\rm{background}}\,{\rm{input}}}.$$

Here *z*_*k*_ denotes the state of the *k* th unit in the background population ${\mathscr{B}}$ and *m*_*ij*_ the weight from unit *j* in the background population to unit *i* in the sampling network. Given the total input field ${h^{\prime} }_{i}$, the neurons in the sampling network change their state deterministically, according to

12 $${F}_{i}({h^{\prime} }_{i})=\Theta ({h^{\prime} }_{i}).$$

Since the units in the background population are mutually unconnected, their average activity 〈*z*_*i*_〉 can be arbitrarily set by adjusting their bias: *b*_*k*_ = *F*^−1^(〈*z*_*k*_〉), where *F*^−1^ denotes the inverse of the logistic activation function:

$${F}^{-1}(\langle z\rangle )=\frac{1}{\beta }\,\log \,\frac{1}{\frac{1}{\langle z\rangle }-1}.$$

Ignoring the actual state of the background population, we can employ the central limit theorem and approximate the background input in the input field ${h^{\prime} }_{i}$ by a normal distribution with mean and variance given by

13 $${\mu }_{i}=\sum _{k\in  {\mathcal B} }\,{m}_{ik}\langle {z}_{k}\rangle ,$$

14 $${\sigma }_{i}^{2}=\sum _{k\in  {\mathcal B} }\,{m}_{ik}^{2}\langle {z}_{k}\rangle \mathrm{(1}-\langle {z}_{k}\rangle \mathrm{).}$$

The total input field can then be written as ${h^{\prime} }_{i}$ = *h*_*i*_ + *ξ*_*i*_ with ${\xi }_{i} \sim {\mathscr{N}}({\mu }_{i},{\sigma }_{i}^{2})$, as in the case of private uncorrelated Gaussian noise. However, note that correlations in input fields ${h^{\prime} }_{i}$ and ${h^{\prime} }_{j}$ in the sampling network arise due to units in the background population projecting to multiple units in the sampling network (〈(*ξ*_*i*_ − *μ*_*i*_)(*ξ*_*j*_ − *μ*_*j*_)〉 does not necessarily vanish for all $i,j\in {\mathscr{S}}$).

For the connections from the background population we use fixed weights and impose Dale’s law, i.e., units are either excitatory *m*_*ij*_ = *w* > 0 ∀*i* or inhibitory *m*_*ij*_ = −*gw* < 0 ∀*i*, with a ratio of excitatory units of $\gamma =|{ {\mathcal B} }_{E}|/| {\mathcal B} |$. Here $w\in {{\mathbb{R}}}^{+}$ denotes the excitatory synaptic weight and $g\in {{\mathbb{R}}}^{+}$ a scaling factor for the inhibitory weights. Each unit $i\in {\mathscr{S}}$ in the sampling network receives exactly $K=\varepsilon N$ inputs from units in the background population. $\varepsilon =K/N\in [0,1]$ is referred to as the connectivity. We do not allow multiple connections between a unit in the sampling network and unit in the background population. Assuming all units in the background population have identical average activity 〈*z*〉, all units in the sampling network receive statistically identical input and the equations for the mean and variance simplify to

15 $$\mu =Kw\,(\gamma -(1-\gamma )g)\langle z\rangle ,$$

16 $${\sigma }^{2}=K{w}^{2}(\gamma +\mathrm{(1}-\gamma ){g}^{2})\langle z\rangle \mathrm{(1}-\langle z\rangle \mathrm{).}$$

We can hence employ the same procedure as in the previous section to relate the strength of the background input to the inverse temperature of a Boltzmann machine.

### Network noise

We now consider a background population of deterministic units projecting to the sampling network. The background population has sparse, random, recurrent connectivity with a fixed indegree. Connections in the background population are realized with the same indegrees *K*, weights *w* and −*gw* and ratio of excitatory inputs *γ* as the connections to the sampling network (cf. Shared noise). The connection matrix of the background population is hence generally asymmetric. As before, we can approximate the additional contribution to the input fields of neurons in the sampling network with a normal distribution, with parameters

17 $${\mu }_{i}=\sum _{k\in  {\mathcal B} }\,{m}_{ik}\langle {z}_{k}\rangle ,$$

18 $${\sigma }_{i}^{2}=\sum _{k\in  {\mathcal B} }\,{m}_{ik}^{2}\langle {z}_{k}\rangle (1-\langle {z}_{k}\rangle )+\sum _{k\ne l}\,{m}_{ik}{m}_{il}{c}_{kl},$$

where the additional term in the input variances arises from correlations *c*_*kl*_ :=〈(*z*_*k*_ − 〈*z*_*k*_〉)(*z*_*k*_ − 〈*z*_*k*_〉)〉 between units in the background population. As in the sampling network we choose the bias to cancel the expected average input from other units in the network for a desired mean activity 〈*z*_*k*_〉. However since the second population exhibits rich dynamics due to its recurrent connectivity the actual average activity will deviate from this value, in particular due to an influence of correlations on the mean activity. We employ an iterative meanfield-theory approach that allows us to compute average activities and average correlations approximately from the statistics of the connectivity. We now shortly summarize this approach following^39^. Note that in the literature a threshold variable *θ*_*i*_ is often used instead the bias *b*_*i*_, which differs in the sign: *b*_*i*_ = −*θ*_*i*_.

For a network of binary units, the joint distribution of network states *p*(*s*) contains all information necessary to statistically describe the network activity, in particular mean activities and correlations. It can be obtained by solving the Master equation of the system, which determines how the probability masses of network states evolve over time in terms of transition probabilities between different states^70^

19 $${\partial }_{t}p({{\rm{s}}}_{i})=\sum _{j}\,p({{\rm{s}}}_{i}|{{\rm{s}}}_{j})p({{\rm{s}}}_{j})-p({{\rm{s}}}_{j}|{{\rm{s}}}_{i})p({{\rm{s}}}_{i}\mathrm{).}$$

The first term describes probability mass moving into state *i* from other states *j* and the second term probability mass moving from state *i* to other states *j*. Since in general, and in particular in large networks, Eq. 19 is too difficult to solve directly, we focus on obtaining equations for first two momenta of *p*(*s*). Starting from the master equation one can derive the following self-consistency equations for the mean activity of units in a homogeneous network by assuming fluctuations around their mean input to be statistically independent^39^:

$${{\rm{\partial }}}_{t}\langle {s}_{i}\rangle +\langle {s}_{i}\rangle =\frac{1}{2}{\rm{e}}{\rm{r}}{\rm{f}}{\rm{c}}(-\,\frac{{\mu }_{i}+{b}_{i}}{\sqrt{2}{\sigma }_{i}})$$

where the *μ*_*i*_ and *σ*_*i*_ are given by Eqs. 17 and 18, respectively. To obtain the average activity in the stationary state, i.e., for ∂_*t*_〈*s*_*i*_〉 = 0, this equation needs to be solved self-consistently since the activity of unit *i* can influence its input statistics (*μ*_*i*_, *σ*_*i*_) through the recurrent connections. By assuming homogeneous excitatory and inhibitory populations, the *N* dimensional problem reduces to a two-dimensional one^39^:

20 $$\langle {s}_{\alpha }\rangle =\frac{1}{2}{\rm{e}}{\rm{r}}{\rm{f}}{\rm{c}}(-\,\frac{{\mu }_{\alpha }+{b}_{\alpha }}{\sqrt{2}{\sigma }_{\alpha }})$$

with $\alpha \in \{E,I\}$. The population-averaged equations for the mean and variance of the input hence are^39^:

21 $${\mu }_{\alpha }=\sum _{\beta }\,{K}_{\alpha \beta }{w}_{\alpha \beta }{s}_{\beta },$$

22 $${\sigma }_{\alpha }^{2}=\sum _{\beta }\,{K}_{\alpha \beta }{w}_{\alpha \beta }^{2}{a}_{\beta }+\sum _{\beta ,\gamma }\,{(Kw)}_{\alpha \beta }{(Kw)}_{\alpha \gamma }{c}_{\beta \gamma },$$

with *K*_*EE*_ = *K*_*IE*_ = *γN*, *K*_*EI*_ = *K*_*II*_ = (1 − *γ*)*N* and *w*_*EE*_ = *w*_*IE*_ = *w*, *w*_*EI*_ = *w*_*II*_ = −*gw*. To derive a self-consistency equation for pairwise correlations from the master equation one linearize the threshold activation function by considering a Gaussian distribution of the input field caused by recurrent inputs. This leads to the following set of linear equations for the population-averaged covariances^39^:

23 $$2{c}_{\alpha \beta }=\sum _{\gamma }\,({\tilde{w}}_{\alpha \gamma }{c}_{\gamma \beta }+{\tilde{w}}_{\beta \gamma }{c}_{\gamma \alpha })+{\tilde{w}}_{\alpha \beta }\frac{{a}_{\beta }}{{N}_{\beta }}+{\tilde{w}}_{\beta \alpha }\frac{{a}_{\alpha }}{{N}_{\alpha }},$$

with

$${c}_{\beta \gamma }=\{\begin{array}{ll}\frac{1}{{N}_{\beta }({N}_{\beta }-\mathrm{1)}}{\sum }_{i,j\in \beta ,i\ne j}{c}_{ij} & {\rm{if}}\,\beta =\gamma \\ \frac{1}{{N}_{\beta }{N}_{\gamma }}{\sum }_{i\in \beta ,j\in \gamma }{c}_{ij} & {\rm{else}}\end{array}$$

The effective population-averaged weights ${\tilde{w}}_{\alpha \beta }$ are defined as:

$${\tilde{w}}_{\alpha \beta }\,:=S({\mu }_{\alpha },{\sigma }_{\alpha }){K}_{\alpha \beta }{w}_{\alpha \beta },$$

with the susceptibility given by $S({\mu }_{\alpha }{\sigma }_{\alpha }):=\frac{1}{\sqrt{2\pi }{\sigma }_{\alpha }}\exp (-\frac{{({\mu }_{\alpha }+{b}_{\alpha })}^{2}}{2{\sigma }_{\alpha }})$^39^. Since the average activity and covariances are mutually dependent, we employ an iterative numerical scheme in which we first determine the stationary activity under the assumption of zero correlations according to Eq. 20. Using this result we compute the population-averaged covariances from Eq. 23 which in turn can be used to improve the estimate for the stationary activity since they influence input statistics according to Eq. 22. We repeat this procedure until the values for population-averaged activities and covariances in two subsequent iterations do not differ significantly any more. The mean activity and correlations in the recurrent background population obtained via this procedure, allows us to compute the input statistics in the sampling network and hence relate the inverse temperature to the mean and variance of the input as in Private noise.

Certain assumptions enter this analytical description of network statistics, which might not be fulfilled in general. The description becomes much more complicated for spiking neuron models with non-linear subthreshhold dynamics like conductance-based neurons in neuromorphic systems^15^. In this case, one can resort to empirically measuring the input statistics for a single isolated neuron given a certain arrangement of background sources (cf. Calibration (spiking networks)). An advantage of this methods is that it is easy and straight forward to implement and will work for any configuration of background populations and sampling networks, allowing for arbitrary neuron models and parameters. However, to estimate the statistics of the input accurately, one needs to collect statistics over a significant amount of time.

### Calibration (binary networks)

The methods discussed above allow us to compute effective inverse temperature *β*_eff_ from the statistics of different background inputs, either additive Gaussian noise, a population of intrinsically stochastic units or a recurrent network of deterministic units. To approximate Boltzmann distributions via samples generated by networks with noise implemented via these alternative methods, we match their (effective) inverse temperatures. A straightforward option is to adjust the noise parameter according to the desired input statistics. While this is possible in the case of additive Gaussian noise for which we can freely adjust *μ*_*i*_ and *σ*_*i*_, it is difficult to achieve in practice for the other methods. We can achieve the same effect by rescaling the weights and biases in the sampling network. The inverse temperature *β* appears as a multiplicative factor in front of weights and biases in the stationary distribution of network states (Eq. 4). Scaling *β* is hence equivalent to scaling all weights and biases by the inverse factor^15,40,71^. An infinite amount of Boltzmann machines hence exists, all differing in weights ($w\to \alpha w,\alpha \in {{\mathbb{R}}}^{+}$), biases (*b* → *αb*) and inverse temperatures (*β* → *β*/*α*), producing statistically identical samples. Given a mean background input *μ*_*i*_ and an effective inverse temperature *β*_eff_(*σ*_*i*_) (cf. Eq. 10) arising from a particular realization of noise sources, we can emulate a Boltzmann machine at inverse temperature *β* by rescaling all weights and biases in the sampling network according to

24 $${b}_{i}\to \beta /{\beta }_{{\rm{eff}}}\,{b}_{i}-{\mu }_{i},$$

25 $${w}_{ij}\to \beta /{\beta }_{{\rm{eff}}}\,{w}_{ij}.$$

This method hence only requires us to adapt weights and biases globally in the sampling network according to the statistics arising from an arbitrary realization of background input.

### Handwritten-digit generation

In the generative task, we measure how well a sampling network with various realizations of background noise can approximate a trained data distribution, in contrast to the random distributions considered in the other simulations. We use contrastive divergence (CD-1^36^) to train a Boltzmann machine on a specific dataset. We consider a dataset consisting of a subset of MNIST digits^72^, downscaled to 12 × 12 pixels and with grayscale values converted to black and white. We select one representative from each class (0…9) and extend the 144 array determining the pixel values with 10 entries for a one-hot encoding of the corresponding class, e.g., for the pattern zero, the last ten entries contain a 1 at the first place and zeros otherwise. These ten 154 dimensional patterns form the prototype dataset. A (noisy) training sample is generated by flipping every pixel from the first 144 entries of a prototype pattern with probability *p*^flip^. After training, the network should represent a particular distribution q^*^ over classes. Training directly on a samples generated according to the class distribution q^*^ will, in general, lead to a different stationary distribution of one-hot readout states *p* generated by the network, since some patterns are more salient then others. For example, by training on equal amounts of patterns of zeros and ones, the network will typically generate more zero states. To nevertheless represent q^*^ with the network, we iteratively train the Boltzmann machine choosing images and labels from a distribution *q* that is adjusted between training sessions (Alg. 1).

**Algorithm 1:**
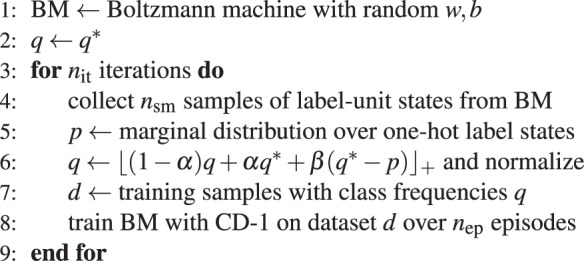
Training a fully visible Boltzmann machine via CD-1 to represent a particular distribution q^*^ over label units with one-hot encoding.

Over many repetitions this procedure will lead to a stationary distribution of classes *p* that closely approximates q^*^.

After training a Boltzmann machine using this approach, we obtain a set of parameters, *w* and *b*, that can be translated to parameters for sampling networks by appropriate rescaling as discussed above. We collect samples from *p* by running the network in the absence of any input and recording the states of all label units.

### Calibration (spiking networks)

Similar as for binary units, we need to match the parameters for spiking sampling networks to their respective counterparts in Boltzmann machines. We use high-rate excitatory and inhibitory inputs to turn the deterministic behavior of a leaky-integrate-and-fire neuron into an effectively stochastic response^15^. However, in contrast to the original publication, we consider current-based synapses for simplicity. Since the calibration is performed on single cell level, we use the identical calibration scheme for the private, shared and network case. For a given configuration of noise sources, we first simulate the noise network with the specified parameters and measure its average firing rate. The corresponding independent Poisson sources are set to fire with the same rate to ensure comparability between the two approaches. The calibrations are then performed by varying the resting potential and recording the average activity of a single cell that is supplied with input from either a noise network or Poisson sources. The private case is calibrated separately in a similar manner. By fitting the logistic function to the activation obtained by this procedure, we obtain two parameters, a shift and a scaling parameter, which are used to translate the synaptic weights from binary units to spiking neurons^15,73^.

## Supplementary information

Supplementary information
