# Supplementary material for: Deterministic networks for probabilistic computing
Source: Sci Rep. 2019 Dec 4;9:18303. doi: 10.1038/s41598-019-54137-7 (PMC6893033; doi:10.1038/s41598-019-54137-7)
Supplement: Supplementary file 1 — Supplementary information [file 41598_2019_54137_MOESM1_ESM.pdf]

Supplementary material for: Deterministic networks for probabilistic computing

Jakob Jordan[1,2,*], **Mihai A. Petrovici**[2,3], **Oliver Breitwieser**[3], **Johannes Schemmel**[3], **Karlheinz Meier**[3], **Markus Diesmann**[1,4,5], **and Tom Tetzlaff**[1]

[1]Institute of Neuroscience and Medicine (INM-6) and Institute for Advanced Simulation (IAS-6) and JARA Institute Brain-Structure-Function Relationships (JBI-1 / INM-10), Jülich Research Centre, Jülich, Germany
[2]Department of Physiology, University of Bern, Bern, Switzerland
[3]Kirchhoff Institute for Physics, Ruprecht-Karls-University Heidelberg, Heidelberg, Germany
[4]Department of Psychiatry, Psychotherapy and Psychosomatics, Medical Faculty, RWTH Aachen University, Aachen, Germany
[5]Department of Physics, Faculty 1, RWTH Aachen University, Aachen, Germany
[*]jordan@pyl.unibe.ch

ABSTRACT

Pairwise input correlations

Here we show how the covariance C^{in}_{kl} between the input fields of two units k and l receiving inputs from a pool of sources can be decomposed into a part arising from shared inputs and another from activity correlations. The input field for a single unit is given by Eq. 2 in the main manuscript and hence:

$$\begin{aligned}
C^{\mathrm{in}}_{kl} &= \langle h_k h_l \rangle - \langle h_k \rangle \langle h_l \rangle \\
&= \left\langle \left(\sum_i^K w_{ki} s_i + b_k \right) \left(\sum_j^K w_{lj} s_j + b_l \right) \right\rangle - \langle h_k \rangle \langle h_l \rangle \\
&= \sum_i^K w_{ki} w_{li} A_i + \sum_i^K \sum_{j \neq i}^K w_{ki} w_{lj} C_{ij} \\
&= C^{\mathrm{in}}_{\mathrm{shared},kl} + C^{\mathrm{in}}_{\mathrm{corr},kl} \, .
\end{aligned}$$

We introduced the auto- and crosscovariances $A_i = \langle s_i^2 \rangle - \langle s_i \rangle^2$ and $C_{ij} = \langle s_i s_j \rangle - \langle s_i \rangle \langle s_j \rangle$ of the activities s_i and s_j of presynaptic neurons i and j, respectively. If Dale's law is respected and the sign of all outgoing connections of a sources is unique, i.e. $\mathrm{sign}(w_{ki}) = \mathrm{sign}(w_{li}),\ \forall k,l,i$, the first term is always positive ($C^{\mathrm{in}}_{\mathrm{shared},kl} > 0$). For a pool of independently active presynaptic neurons, $C_{ij} = 0$ by definition and the second term in the input correlations hence vanishes ($C^{\mathrm{in}}_{\mathrm{corr},kl} = 0\ \forall k,l$). The total input correlation is therefore always positive and determined by the number of shared sources. If the presynaptic sources are units in a recurrently connected network, their pairwise correlation is in general non-zero ($C_{ij} \neq 0$). In particular, in sparsely connected networks with sufficient inhibition, correlations arrange such that $C^{\mathrm{in}}_{\mathrm{corr},kl} \approx -C^{\mathrm{in}}_{\mathrm{shared},kl}$, leading to small remaining pairwise input correlations, $C^{\mathrm{in}}_{kl} \approx 0$[1,2].

Sampling error depends on number of noise inputs per sampling unit

To closely approximate the effect of Gaussian noise on the input field, one needs a large number K of background inputs per sampling unit. Here, we scale the number of noise sources K per sampling unit, while also scaling the total number N of noise sources to keep their ratio constant. This allows us to investigate the impact of K without altering the amount of shared-input correlations. In addition to the three cases considered in main manuscript (private, shared, network noise), we additionally consider the case of a separate pool of noise sources for each sampling unit ("discrete"), where shared-input correlations are absent.

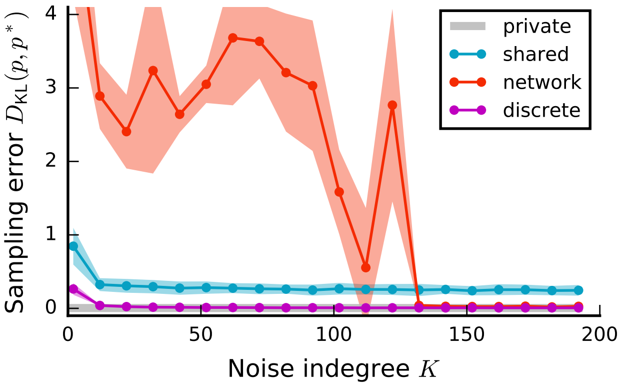

Figure 1. Sampling error $D_{KL}(p, p^*)$ as a function of the number of background inputs K per sampling unit. Error bands indicate mean $\pm$ SEM over 5 random network realizations. Magenta ("discrete") uses K separate sources for each sampling unit. Sampling duration $T = 10^5$ ms. Connectivity constant $K/N = 0.9$. Remaining parameters as in Fig. 2 in the main manuscript.

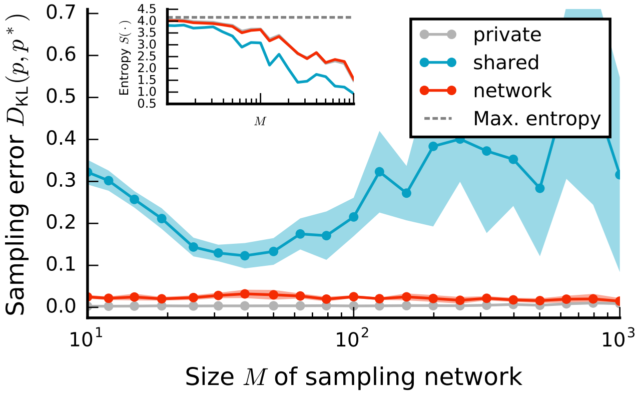

Figure 2. Same as Fig. 7 in the main manuscript, but with constant average weight in sampling networks: $\mu_{BM} = -0.15$.

For small K, the input distribution is strongly discretized and does not approximate Gaussian well, reflected in a large sampling error for very small K for the discrete and shared case (Fig. 1). As we increase K, the sampling error decreases rapidly for the discrete case, and drops to the same level as Gaussian noise at about 50 inputs. For the shared case, the error decreases as well as we increase K, but is limited from below by sampling error introduced by shared-input correlations. For the network case, the sampling error is very large for small K as the network dynamics lock into a fixed point. However, for $K > 130$, the sampling error for the network case drops almost to the level of Gaussian noise.

Small, recurrent networks can supply large sampling networks with noise – no weight scaling

In Fig. 7 in the main manuscript we scaled the weights in the sampling network with the size M of the sampling network as $1/\sqrt{M}$. Ignoring the influence of cross-correlations, this scaling keeps the variances of the input distribution arising from recurrent connections in the sampling network constant. Effectively this leads to approximately constant entropy for a large range of sampling network sizes.

If we do not scale the weights as above when increasing the size of the sampling network, the input variance increases and the relative noise strength hence decreases, leading to an effectively stronger coupled sampling network. This strongly decreases the entropy of the sampled distribution (Fig. 2, inset). Despite the decrease in entropy, the sampling errors for the private and network cases stay approximately constant (Fig. 2). For the shared case, the sampling error initially decreases due to the strengthened effective feedback that suppresses shared-input correlations arising from the limited pool of background sources (cf. **Small, recurrent networks can supply large sampling networks with noise**). As the size of the sampling network increases the sampling error increases again from about $M = 40$. This is most likely caused by the decrease in the relative noise

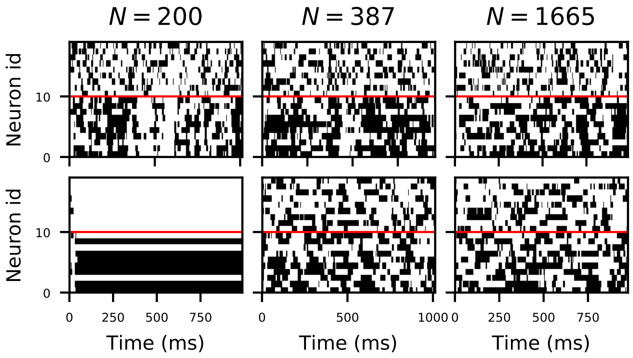

Figure 3. Example activity of sampling units (id $0-9$) and noise sources (id $10-19$) for different number N of noise sources. Top row: shared noise, bottom row: network noise.

strength and the sampling dynamics hence becoming too slow to approximate the target distribution in the finite sampling duration considered here.

Synchronization of noise networks for small network sizes

Fig. 3 illustrates the activity in the noise pool for network and shared noise for different number of noise sources N. If the network becomes too densely connected, the activity of the noise network gets stuck in a fixed point which also leads to a fixed state in the sampling units. This generally causes the large errors for small noise networks.

Simulation details

Tab. 1, 2, 3, 4, 5 summarize the binary network model and parameters.

Tab. 6, 7, 8, 9 summarize the spiking network model and parameters. Simulations carried out with NEST 2.10[3].

References

1. Renart, A. et al. The asynchronous state in cortical circuits. science **327**, 587–590 (2010).
2. Tetzlaff, T., Helias, M., Einevoll, G. T. & Diesmann, M. Decorrelation of neural-network activity by inhibitory feedback. PLoS Comput. Biol **8**, e1002596 (2012).
3. Bos, H. et al. Nest 2.10.0 (2015). URL https://doi.org/10.5281/zenodo.44222. DOI 10.5281/zenodo.44222.
4. Nordlie, E., Gewaltig, M.-O. & Plesser, H. E. Towards reproducible descriptions of neuronal network models. PLoS computational biology **5**, e1000456 (2009).

A	Model summary	
Populations	One	
Topology	—	
Connectivity	All-to-all	
Neuron model	Stochastic binary units	
Channel models	—	
Synapse model	—	
Plasticity	—	
External input	—	
Measurements	Binary states of m units	
**B**	**Populations**	
**Name**	**Elements**	**Size**
Sampling network	Stoch. binary units	M
**C**	**Connectivity**	
**Source**	**Target**	**Pattern**
Sampling network	Sampling network	All-to-all, random weights drawn from Beta distribution, $w_{ij} \sim \text{Beta}(a,b)$, symmetric connections $w_{ij} = w_{ji}$, no self connections $w_{ii} = 0$
**D**	**Neuron model**	
Type	Stochastic binary units	
Dynamics	Transition into state 1 according to probability determined by the activation function $F_i(h_i) = \frac{1}{1+e^{-\beta h_i}}$ with input field $h_i = \sum_j w_{ij} s_j + b_i$.	

Table 1. Description of the sampling network model with intrinsic noise (according to[4]).

A	Model summary	
Populations	One	
Topology	—	
Connectivity	All-to-all	
Neuron model	Stochastic binary units	
Channel models	—	
Synapse model	—	
Plasticity	—	
External input	—	
Measurements	Binary states of m units	
**B**	**Populations**	
**Name**	**Elements**	**Size**
Sampling network	Stoch. binary units	M
**C**	**Connectivity**	
**Source**	**Target**	**Pattern**
Sampling network	Sampling network	All-to-all, random weights drawn from Beta distribution, $w_{ij} \sim \text{Beta}(a,b)$, symmetric connections $w_{ij} = w_{ji}$, no self connections $w_{ii} = 0$
**D**	**Neuron model**	
Type	Stochastic binary units	
Dynamics	Transition into state 1 according to probability determined by the activation function $F_i(h_i) = \frac{1}{2}\text{erfc}\left(\frac{h_i+\mu_i}{\sqrt{2\sigma^2}}\right)$ with input field $h_i = \sum_j w_{ij} s_j + b_i$.	

Table 2. Description of sampling network model with private noise (according to[4]).

A	Model summary
Populations	Three
Topology	—
Connectivity	All-to-all; sparse random with fixed indegree
Neuron model	Stochastic binary units, deterministic binary units
Channel models	—
Synapse model	—
Plasticity	—
External input	—
Measurements	Binary states

B	Populations	
**Name**	**Elements**	**Size**
Sampling network	Det. binary units	M
Background pop. (E)	Stoch. binary units	γN
Background pop. (I)	Stoch. binary units	$(1-\gamma)N$

C	Connectivity	
**Source**	**Target**	**Pattern**
Sampling network	Sampling network	All-to-all, random weights drawn from Beta distribution, $w_{ij} \sim \mathrm{Beta}(a,b)$, symmetric connections $w_{ij} = w_{ji}$, no self connections $w_{ii} = 0$
Background pop. (E)	Sampling network	Random convergent $\gamma K \to 1$, weight w
Background pop. (I)	Sampling network	Random convergent $(1-\gamma)K \to 1$, weight $-gw$

D	Neuron model
Type<br>Dynamics	Stochastic binary units<br>Transition into state 1 according to probability determined by the activation function $F_i(h_i) = \frac{1}{1+e^{-\beta h_i}}$ with input field $h_i = \sum_j w_{ij} s_j + b_i$.
Type<br>Dynamics	Deterministic binary units<br>Transition into state 1 according to the activation function $F_i(h_i) = \Theta(h_i)$ with input field $h_i = \sum_j w_{ij} s_j + b_i$.

E	Measurements
Binary states of m units from sampling network	

Table 3. Description of sampling network model with shared noise (according to[4]).

A	Model summary
Populations	Three
Topology	—
Connectivity	All-to-all; sparse random with fixed indegree
Neuron model	Deterministic binary units
Channel models	—
Synapse model	—
Plasticity	—
External input	—
Measurements	Binary states

B	Populations	
**Name**	**Elements**	**Size**
Sampling network	Det. binary units	M
Background pop. (E)	Det. binary units	γN
Background pop. (I)	Det. binary units	$(1-\gamma)N$

C	Connectivity	
**Source**	**Target**	**Pattern**
Sampling network	Sampling network	All-to-all, random weights drawn from Beta distribution, $w_{ij} \sim \mathrm{Beta}(a,b)$, symmetric connections $w_{ij} = w_{ji}$, no self connections $w_{ii} = 0$
Background pop. (E)	Sampling network	Random convergent $\gamma K \to 1$, weight w
Background pop. (I)	Sampling network	Random convergent $(1-\gamma)K \to 1$, weight $-gw$
Background pop. (E)	Background pop. (E)	Random convergent $\gamma K \to 1$, weight w
Background pop. (E)	Background pop. (I)	Random convergent $\gamma K \to 1$, weight w
Background pop. (I)	Background pop. (E)	Random convergent $(1-\gamma)K \to 1$, weight $-gw$
Background pop. (I)	Background pop. (I)	Random convergent $(1-\gamma)K \to 1$, weight $-gw$

D	Neuron model
Type<br>Dynamics	Deterministic binary units<br>Transition into state 1 according to the activation function $F_i(h_i) = \Theta(h_i)$ with input field $h_i = \sum_j w_{ij} s_j + b_i$.

E	Measurements
Binary states of m units from sampling network	

Table 4. Description of sampling network model with network noise (according to[4]).

**B**	**Populations**	
**Name**	**Values**	
M	100^*	
N	222^*	
γ	0.3	
**C**	**Connectivity**	
**Name**	**Values**	
a	2	
b	2	
μ_{BM}	0.0	
K	200	
w	0.3	
g	8	
**D**	**Neuron model**	
**Name**	**Values**	
β	1^*	
μ	0	
σ	from β via Eq. 10	
**E**	**Measurements**	
**Name**	**Values**	
m	6	
	**Miscellaneous**	
**Name**	**Values**	**Description**
$\bar{s}$	0.4	Average activity in sampling networks
$\bar{z}$	0.3	Average activity in background population
T_{sim}	$10^5\,\mathrm{ms}^*$	Simulation time
T_{warmup}	$500\,\mathrm{ms}$	Warmup time (ignored during analysis)
τ	$10\,\mathrm{ms}$	Average inter-update interval

Table 5. Parameters for binary network simulations (according to[4]). Stars indicate default values.

A	Model summary	
Populations	One	
Topology	—	
Connectivity	All-to-all	
Neuron model	Leaky integrate-and-fire (LIF)	
Channel models	—	
Synapse model	Exponentially decaying currents, fixed delays	
Plasticity	—	
External input	Poisson-distributed spike trains	
Measurements	Spikes	

B	Populations	
**Name**	**Elements**	**Size**
Sampling network	LIF neuron	M

C	Connectivity	
**Source**	**Target**	**Pattern**
Sampling network	Sampling network	All-to-all, random weights drawn from Beta distribution, $w_{ij} \sim \mathrm{Beta}(a,b)$, symmetric connections $w_{ij} = w_{ji}$, no self connections $w_{ii} = 0$, translation from binary-unit domain to spiking neurons via constant calibration factors (see Sec. in the main manuscript)

D	Neuron and synapse model
Type	Leaky integrate-and-fire, exponential currents
Subthreshold dynamics	Subthreshold dynamics ($t \notin (t^*, t^* + \tau_{\mathrm{ref}})$):<br>$C_{\mathrm{m}} \frac{\mathrm{d}}{\mathrm{d}t} V(t) = -g_{\mathrm{L}}(V(t) - V_{\mathrm{rest}}) + I_{\mathrm{syn}}(t)$<br>Reset and refractoriness ($t \in (t^*, t^* + \tau_{\mathrm{ref}})$):<br>$V(t) = V_{\mathrm{reset}}$
Current dynamics	$\tau_{\mathrm{syn}} \frac{\mathrm{d}}{\mathrm{d}t} I_{\mathrm{syn}}(t) = -I_{\mathrm{syn}}(t) + \sum_{i,k} J \delta(t - t_i^k - d)$<br>Here the sum over i runs over all presynaptic neurons and the sum over k over all spike times of the respective neuron i
Spiking	If $V(t^*-) < V_{\mathrm{th}} \wedge V(t^*+) \geq V_{\mathrm{th}}$:<br>emit spike with time stamp t^*

E	Measurements
Spike trains recorded from m neurons from the sampling network	

F	External input
Per neuron, one private excitatory and one inhibitory Poisson source with rate ν_{ex} and ν_{in}, respectively.	

Table 6. Description of spiking sampling network model with private noise (according to[4]).

A	Model summary
Populations	One
Topology	—
Connectivity	All-to-all; sparse random with fixed indegree
Neuron model	Leaky integrate-and-fire (LIF)
Channel models	—
Synapse model	Exponentially decaying currents, fixed delays
Plasticity	—
External input	Poisson-distributed spike trains
Measurements	Spikes

B		Populations
**Name**	**Elements**	**Size**
Sampling network	LIF neuron	M

C		Connectivity
**Source**	**Target**	**Pattern**
Sampling network	Sampling network	All-to-all, random weights drawn from Beta distribution, $w_{ij} \sim \text{Beta}(a,b)$, symmetric connections $w_{ij} = w_{ji}$, no self connections $w_{ii} = 0$, translation from binary-unit domain to spiking neurons via constant calibration factors (see Sec. in the main manuscript)

D	Neuron and synapse model
See Tab. 6.	

E	Measurements
See Tab. 6.	

F	External input
Per neuron, γK excitatory and $(1-\gamma)K$ inhibitory Poisson sources with weight J, rate $\tilde{\nu}_{\text{ex}}$ and weight $-gJ$, rate $\tilde{\nu}_{\text{in}}$, respectively. Excitatory and inhibitory inputs randomly chosen from a common pool of γN and $(1-\gamma)N$ units, respectively.	

Table 7. Description of spiking sampling network model with shared noise (according to[4]).

A	Model summary	
Populations	Three	
Topology	—	
Connectivity	All-to-all; sparse random with fixed indegree	
Neuron model	Leaky integrate-and-fire (LIF)	
Channel models	—	
Synapse model	Exponentially decaying currents, fixed delays	
Plasticity	—	
External input	Resting potential above firing threshold in background populations	
Measurements	Spikes	
**B**	**Populations**	
**Name**	**Elements**	**Size**
Sampling network	LIF neuron	M
Background pop. (E)	LIF neuron	γN
Background pop. (I)	LIF neuron	$(1-\gamma)N$
**C**	**Connectivity**	
**Source**	**Target**	**Pattern**
Sampling network	Sampling network	All-to-all, random weights drawn from Beta distribution, $w_{ij} \sim \mathrm{Beta}(a,b)$, symmetric connections $w_{ij} = w_{ji}$, no self connections $w_{ii} = 0$, translation from binary-unit domain to spiking neurons via constant calibration factors (see Sec. in the main manuscript)
Background pop. (E)	Sampling network	Random convergent, $\gamma K \to 1$, weight w, delay d
Background pop. (I)	Sampling network	Random convergent, $(1-\gamma)K \to 1$, weight $-gw$, delay d
Background pop. (E)	Background pop. (E)	Random convergent, $\gamma K \to 1$, weight w, delay d
Background pop. (E)	Background pop. (I)	Random convergent, $\gamma K \to 1$, weight w, delay d
Background pop. (I)	Background pop. (E)	Random convergent, $(1-\gamma)K \to 1$, weight $-gw$, delay d
Background pop. (I)	Background pop. (I)	Random convergent, $(1-\gamma)K \to 1$, weight $-gw$, delay d
**D**	**Neuron and synapse model**	
See Tab. 6.		
**E**	**Measurements**	
See Tab. 6.		

Table 8. Description of spiking sampling network model with network noise (according to[4]).

**B**	**Populations**
See Tab. 5.	

**C**	**Connectivity**
**Name**	**Values**
a	2
b	2
K	1000
J	0.002 nA (0.0635 nA)
g	2
d	0.1 ms (1.0 ms)

**D**	**Neuron model**
**Name**	**Values**
τ_{ref}	10.0 ms (0.1 ms)
τ_{syn}	10.0 ms (5.0 ms)
C_{m}	0.2 nF (1.0 nF)
g_{L}	2.0 µS (0.05 µS)
V_{rest}	−50.00 mV (−40.00 mV)
V_{reset}	−50.01 mV (−60.00 mV)
V_{th}	−50.00 mV

**E**	**Measurements**
**Name**	**Values**
m	10

	**Miscellaneous**	
**Name**	**Values**	**Description**
T_{sim}	10^7 ms	Simulation time
T_{warmup}	10^3 ms	Warmup time (ignored during analysis)

**F**	**External input**
**Name**	**Values**
ν_{ex}	10 kHz
ν_{in}	10 kHz
$\tilde{\nu}_{\text{ex}}$	4.4 ± 0.1 Hz
$\tilde{\nu}_{\text{in}}$	4.4 ± 0.1 Hz

Table 9. Table of parameters for spiking network simulations (according to[4]). Values without parantheses are for the sampling network, values in parantheses for the noise network.
